# An approach to data collection in compassionate use/managed access

**DOI:** 10.3389/fphar.2022.1095860

**Published:** 2022-12-20

**Authors:** Séverine Sarp, Ramona Reichenbach, Paul Aliu

**Affiliations:** Chief Medical Office, Novartis Pharma AG, Basel, Switzerland

**Keywords:** compassionate use of drugs, patient access, managed access, real-world data, patient-centered care, patients’ benefits

## Abstract

Compassionate Use (CU)/Managed Access programs provide access to locally unapproved medicines. As these programs become more global and involve a broader range of products, determining whether patients derive benefit from treatment could provide insights into therapeutic use in a real-word setting with diverse pools of patients. CU primary purpose is to provide treatment and it is not targeting research. However, it is increasingly considered as a source of real-world data. In the absence of a harmonized framework on CU data collection, Novartis developed a company-wide guidance to collect baseline patient data and prospective follow-up information at product resupply. Although this approach has recently been implemented and utilization of this data has been mainly internal to the company so far, the prospective collection of key efficacy parameters in patients receiving therapies *via* CU could potentially be used as a supportive set of information collected in a real-world setting to be submitted in addition to clinical trial data, if not as a main source of data for regulatory submission.

## Introduction

Clinical trials are the channel through which patients access investigational or otherwise unapproved medicines. However, there are patients with serious or life-threatening diseases for whom enrollment in a trial is impossible and all comparable and available treatment options have been exhausted. This scenario is covered by regulations adressing CU in many countries to allow access to unapproved medicines (Aliu et al., 2021). The terminology for these access channel programs varies greatly and can be referred to as “compassionate use,” “managed access,” “expanded access,” “named patient supply,” “special access,” or similar terms (Kimberly et al., 2017; Aliu et al., 2021; Polak et al., 2022a).

In the context of CU, patient data collection can be performed prospectively or retrospectively to assess whether patients are deriving benefit from treatment. We present here the approach recently implemented by Novartis to collect baseline patient parameters and prospectively acquire minimum, key efficacy information at product resupply for selected products and indications, as opposed to the retrospective medical chart review generally used to analyze CU data (Vassar and Holzmann, 2013; Vannucchi et al., 2021).

## What do country regulations say about data collection in compassionate use?

Country regulatory frameworks on data collection range from limited to non-existent and no global guidance has been developed on what type of data should be collected in the context of CU. Nevertheless, more Health Authorities (HAs) are progressively becoming more open to the collection of minimum and meaningful patient data. Good examples include the United States Food and Drug Administration (FDA) Expanded Access (United States Food and Drug Administration Expanded Access, 2022), Health Canada’s Special Access Programme (Health Canada’s Special Access Programme: Overview, 2022), the Italian Compassionate Use Programs (Italian Medicines Agency, Farmaci a uso compassionevole, 2017), and the United Kingdom (UK) Medicines and Healthcare products Regulatory Agency (MHRA)’s Early Access to Medicines Scheme (EAMS) (United Kingdom Medicines and Healthcare products Regulatory Agency EAMS, 2022). In the UK, the option exists to collect limited data under the EAMS scheme without the need for clinical trial approval or ethics review, and to use the data for the technology appraisals of the National Institute for Health and Care Excellence (NICE) (Polak et al., 2022b).

In the European Union overall, despite Article 83 of the European Regulation EC 726/2004 providing general common CU criteria, the article does not mention the concept of data collection. Member States develop and implement their own local CU procedures and, consequently, guidance on data collection during CU differs broadly across Member States, including the type of data which can be collected and how it can be utilised (Polak et al., 2020; Polak et al., 2022a).

In general, based on our experience at Novartis, existing CU regulations typically do not address the data collection concept and there is variety of guidance and interpretation provided, sometimes even within the same country. For instance, in Italy, collection of safety and efficacy data is possible for both individual and cohort access subject to ethics committee approval (Italian Medicines Agency, Farmaci a uso compassionevole, 2017). In Germany however, a mixed approach on data collection is generally taken and depends on the products, indications, or programs in scope (German Federal Institute for Drugs and Medical Devices, “Compassionate Use” Programmes, 2022). Furthermore, in France, the Accès Compassionel (AAC) for individual access usually does not allow data collection by the manufacturer; nevertheless, under Accès Précoce (AAP), data collection can be performed and even utilized in a future reimbursement dossier. In this context, a protocol for therapeutic use and monitoring of patients (PUT) provides a framework for the collection of data which relates to efficacy, adverse events, the actual conditions of use, as well as the characteristics of the population benefiting from treatment (Légifrance Décret, 2022). However, apart from these limited country examples, we recognize that both HAs and legislators in many parts of the world are largely silent on the topic of data collection in CU.

Overall, no global guidance exists on what type of data should or can be collected in CU and in-country regulations vary greatly. Based on our experience, a well-defined regulatory framework strengthens the CU process in a country, as it will provide direction to all involved stakeholders, enable faster patient access, and ensure that key ethical standards and adequate patient safeguards are implemented. Therefore, a robust in-country CU regulatory framework should provide clarity on the collection of patient outcome data for evaluation and the type of data which can be collected, in line with the applicable local data protection or privacy regulations. While CU is not clinical research, collection of data, especially in rare diseases, would contribute to increasing knowledge on the products in these indications. Importantly, collection of patient data, as well as receiving treatment via CU, require valid informed consent in accordance with applicable laws and regulations (e.g., General Data Protection Regulation in the European Union), and is obtained by the treating physician requesting the product through CU. Informed consent ensures that patients understand all known risks and benefits of treatment. In the context of data collection, patients have to give consent to collection of follow-up information at the time of resupply, so as to understand how they are deriving benefit from treatment, if applicable for the product and indication in scope. As real-world data (RWD) collection in CU programs evolves, and as such programs potentially include more complex interventions, we anticipate that consenting and other required patient safeguards will also need to evolve.

## Why is data collection relevant in compassionate use?

Well-designed, ethically sound clinical trials are the basis for solid scientific evaluations of new medicines and therapies. However, based on our experience at Novartis, data collected from CU can represent an important complement to clinical trial data, even when recognizing some weaknesses such as the lack of randomisation and data quality issues (Polak et al., 2020; Rozenberg and Greenbaum, 2020). However, considering that the primary intent of CU is to provide treatment, collection of data during CU does not qualify as, nor replace, clinical trials.

For advanced therapeutics in rare diseases with small patient populations, there is a regulatory requirement for longer-term patient follow-up. This is illustrated in the FDA guidance for industry on follow-up after administration of human gene therapies which recommends long-term observations of genetic effects and delayed adverse event identification to mitigate the long-term risks to the patients (United States Food and Drug Administration, 2020).

There is also an increasing use of targeted therapies across multiple indications. An example is the dabrafenib/trametinib combination, which is provided for a high number of indications *via* CU and was recently FDA approved for first tumor-agnostic indication for BRAF V600E solid tumors (Novartis, 2022).

We also note that pharmaceutical companies and HAs are more regularly including RWD from CU in regulatory submissions (Polak et al., 2020; Polak et al., 2022a). For instance, in April 2022, the FDA granted accelerated approval to alpelisib for severe manifestations of PIK3CA-related overgrowth spectrum (PROS) based on RWD from a single-arm study on patients who received the product as part of a CU program (US Food and Drug Administration, 2022). This study was a retrospective medical chart review, involving a more comprehensive collection of data in the CU setting, as compared to the prospective collection of minimal, key efficacy parameters, which is the main focus of this article. Overall, an integrated approach to evidence generation, which includes the use of data from both clinical trials and real-world settings, offers an interesting model as it provides a holistic picture to clinical researchers and treating physicians, benefits patients by generating value-based outcomes which translate into clinically meaningful effects in the real world, and addresses the increasing RWD demands of HAs (Olson, 2018). However, pharmaceutical companies often face challenges when planning to use data coming from CU and might therefore hesitate to collect this information due to the absence of a clear regulatory framework, concerns about data quality, validation, and credibility, and the prospect of a safety finding reported in an uncontrolled setting impacting ongoing clinical trials. Novartis does see its CU programs and the collection of minimal efficacy data at product resupply for some selected products and indications as entirely complementary to the clinical trials for the related products and indications, leveraging the limitations which the specific circumstances of the collection of this data might bring. In addition, safety information on CU programs is always collected via our dedicated safety database, as performed for all clinical trials and in post-marketing.

## What are the objectives of data collection in compassionate use?

While safety reporting is a matter of country regulatory requirements and has always been part of responsible CU program management, the data collection discussed here is not required for all programs and not a prerequisite to product resupply by Novartis. In fact, the information collected is not evaluated during resupply approval, since this decision is based solely on the physician’s confirmation that their patient is benefiting from treatment. The main objective of data collection is rather to characterize how the patient population in scope is globally deriving benefit, for instance by assessing the evolution of the patients’ status or the additional concomitant treatments needed.

In particular, the collected data can enable a better understanding of therapeutic use in more diverse pools of patients. This is especially relevant for specific patient sub-groups not fulfilling the criteria for trial enrollment or coming from world regions not covered in the related clinical trials. This diversity is most pertinent for rare diseases, where every data point is important to increase the healthcare community’s knowledge on the disease, due to the small number of patients affected. Specifically in these precise circumstances, data collected during CU can therefore supplement clinical trial data. Moreover, collection of data during CU can also be very significant in diseases for which trials might not be under way. Finally, such data may support evaluation and improvement of the CU program itself.

Ultimately, the collected data might help the medical community, regulators, and pharmaceutical industry to better understand the risk-benefit profile of medicines in a setting closer to real-world clinical practice.

Concretely, in terms of the framework which has been put in place by Novartis for prospective collection of data at product resupply, the information collected until now was not yet used as supportive data in a regulatory dossier. However, based on our experience so far, the collection of key efficacy parameters in patients for whom product resupply is requested by their physicians could be used in the future as a supportive set of data collected in a real-world setting, in addition to clinical trial data, if not as main evidence and pivotal source of data for regulatory submission. Results could also be shared with the medical community via peer-reviewed publications.

## How is data collection operationally performed in compassionate use?

Novartis uses an end-to-end online system accessible to all physicians worldwide for the receipt of the initial patient request (Aliu et al., 2022). Once patients are on treatment, physicians can also request product resupply *via* this system for patients deriving benefit, at which timepoint key follow-up efficacy parameters on the patients are captured for specific compounds and indications, according to the principles outlined in Table 1. The rationale for these principles is not to overburden the physician and to only focus on information readily available as part of standard clinical care for the indication in scope.

**TABLE 1:** Principles applied to prospective data collection at product resupply in the Novartis online system.

Collection of prospective, minimal efficacy data at resupply	
1	Targets a maximum of five questions related to key efficacy parameters, which are complementary between baseline and resupply
2	Information easily available to treating physicians as part of the routine care and monitoring for the indication in scope, i.e. no additional medical tests required
3	Only requires a predefined answer from a drop-down list, a numeric value (e.g. a relevant laboratory value that is a surrogate marker of clinical benefit) or a yes/no response
4	No free text responses
5	Use of plain language which can be easily understood by treating physicians around the world
6	Focuses on qualifying or quantifying the benefit derived by the patients on treatment
7	Aligns with the key endpoints in the pivotal clinical trial for the product (when relevant and feasible)

Often, the selected resupply questions are based on the previously studied key endpoints of the clinical trials for the indication. An example of questions used at CU product resupply in the sickle cell disease indication is presented in Table 2.

**TABLE 2:** Example of complementary baseline and resupply questions for sickle cell disease (SCD).

Baseline questions	Resupply questions
What is the total number of vaso-occlusive crises (VOCs) managed at home in the past 12 months?	What is the total number of VOCs managed at home in the past 6 months?
What is the total number of VOCs resulting in a healthcare facility visit in the past 12 months?	What is the total number of VOCs resulting in a healthcare facility visit in the past 6 months?
Has the patient taken opioids for VOCs in the past 12 months?	Has the patient reported any opioid usage for VOCs in the past 6 months?
If the patient has leg ulcer due to SCD, what is the grade?	If the patient has leg ulcer due to SCD, what is the grade in the past 3 months (prior to current resupply request)?
If the patient has proteinuria due to SCD, what is the grade?	If the patient has proteinuria due to SCD, what is the grade in the past 3 months (prior to current resupply request)?

If necessary, additional patient data collection can further be performed retrospectively when physicians are asked to provide information based on patients’ retrospective chart reviews (Vassar and Holzmann, 2013; Vannucchi et al., 2021; US Food and Drug Administration, 2022). Lastly, prospective data collection *via* registries, especially in rare diseases and with one-time therapies, is another tool for collecting additional long-term patient data, as illustrated by the onasemnogene abeparvovec-xioi (Zolgensma) spinal muscular atrophy disease registry (Clinicaltrials.gov, 2022).

## Final remarks

The approach taken by Novartis to collect CU data prospectively at product resupply will contribute to gathering more knowledge on the use and benefits of providing unapproved medicines in a patient population that has exhausted all other available treatment options.

However, prospective collection of data at product resupply also has limitations. One is the inherent bias that information will only be obtained for patients assessed by their physician as deriving benefit from the treatment, hence the request for a product resupply. Nevertheless, collection of data in the subset of patients who are benefiting from treatment and for whom information is collected at resupply can help inform how patients are responding and how key efficacy parameters evolve in this context.

In general, while there may well be indicators of positive efficacy from the data collected at the product resupply timepoint, there are some instances where a full retrospective chart review of CU data is indicated. For example, such analysis might help to further demonstrate efficacy for HA approval (European Medicines Agency, 2017; US Food and Drug Administration, 2022). Overall, HAs and pharmaceutical companies are gradually using more data from sources outside of clinical trials. This RWD represents an opportunity to acquire more knowledge on specific diseases or patient populations in the real-world treatment setting.

In summary, prospective collection of selected efficacy parameters in CU can greatly contribute to the understanding of how patients are globally responding to the provided treatments. To fully realize the benefit of such data collection, harmonized regulations and guidance on CU data collection and associated valid patient consent would greatly help to ensure consistent, reliable, and responsible data collection and analysis of the information obtained in this real-world setting.

## Data Availability

The original contributions presented in the study are included in the article/Supplementary Material, further inquiries can be directed to the corresponding author.
